# Automated AMBU Ventilator With Negative Pressure Headbox and Transporting Capsule for COVID-19 Patient Transfer

**DOI:** 10.3389/frobt.2020.621580

**Published:** 2021-01-29

**Authors:** Arnon Jumlongkul

**Affiliations:** School of Medicine, Mae Fah Luang University, Chiang Rai, Thailand

**Keywords:** automated AMBU, COVID-19, HEPA filter, negative pressure air, transporting capsule

## Abstract

**Purpose:** It is now clear that the COVID-19 viruses can be transferred via airborne transmission. The objective of this study was to attempt the design and fabrication of an AMBU ventilator with a negative pressure headbox linked to a negative pressure transporting capsule, which could provide a low-cost construction, flexible usage unit, and also airborne prevention that could be manufactured without a high level of technology.

**Method:** The machine consists of an automated AMBU bag ventilator, a negative pressure headbox, and a transporting capsule. The function and working duration of each component were tested.

**Results:** The two main settings of the ventilator include an active mode that can be set at the time range of 0 s–9 h 59 min 59 s and a resting mode, which could work continuously for 24 h. The blower motor and battery system, which were used to power the ventilator, create negative air pressure within the headbox, and the transporting capsule, could run for at least 2 h without being recharged. The transporting capsule was able to create an air change rate of 21.76 ACH with-10 Pa internal pressure.

**Conclusion:** This automated AMBU ventilator allowed flow rate, rhythm, and volume of oxygen to be set. The hazardous expired air was treated by a HEPA filter. The patient’s transporting capsule is of a compact size and incorporates the air treatment systems. Further development of this machine should focus on how to link seamlessly with imaging technology, to verify standardization, to test using human subjects, and then to be the commercialized.

## Introduction

The COVID-19 situation has resulted in the changing of many standard intubation and transportation procedures. Viruses can easily transmit via the airborne route and so many scientists have tried to create medical devices that can restrict the spread of COVID-19. Certain issues have to considered for the transport of critically ill COVID-19 patients, these include transport equipment pre-arrangements (e.g., portable ventilator, bag-valve-mask with oxygen tubing, infusion pump), preparations before transport (e.g., team coordination, limiting to essential medical personnel), transport process (e.g., appropriate PPE, droplet precautions), then after arrival, post-transfer decontamination (e.g., a housekeeping team equipment as well as appropriate PPE to remove contaminations from the transfer route, patient’s room, transport ambulance, and also preparing for the next mission), respectively (Yousuf et al., 2020). The concept of a mechanical ventilator started in the 14th century and the use of a positive-pressure mechanical ventilator became common around 1940. A typical mechanical ventilator consists of a control unit, blender, valves/turbine, and sensors. The construction of the ventilation mode should involve three elements, including, ventilator breath control variable (volume and pressure control), breath sequence, and targeting scheme (Dellaca’ et al., 2017). In mass casualty cases, low-cost portable mechanical ventilators have proved to be essential. The ventilator mainly assists breathing using compression of a conventional bag valve mask (BVM) with an electric motor, an over-pressurization alarm system, and microcontroller board (Al Husseini et al., 2010). Due to the lack of adequate supplies of mechanical ventilators, exposed by the COVID-19 pandemic, a new portable ventilator design is suggested. The concept of this ventilator is the addition of a DC motor as a primary air compressor. Additional components also include a temperature sensor, heating resistor, pressure sensor, battery supply, audio alarms, LCD, and a start/stop system (El Majid et al., 2020). This provides the energy to overcome the BVM/air tank resistance in our prototypes, the estimation of tidal volume is then measured indirectly.

One of the interesting technologies that protect against COVID-19 spread is the use of negative pressure systems. According to the recommendations of the World Health Organization, probable or confirmed COVID-19 cases should avoid being moved or transported out of their area unless absolutely necessary, at which time availability of portable equipment becomes essential. Adequate ventilation is considered to be 6–12 air changes per hour (ACH) with a negative pressure difference of at least 2.5 Pa. Installation of exhaust fans and high-efficiency particulate air (HEPA) filters is required for environmental control (World Health Organization, 2020). For patient-care areas, the time required for airborne-contaminant removal by 99%, with 99.9% efficiency when the airflow is set as 12 ACH, is 23 min and 35 min, respectively, (Center for Disease Control and Prevention, 2019). One technique, which has been used to protect us from hazardous and dangerous situations, is the insulated patient transport capsule. Such systems can help protect medical personnel and patients against chemical, biological, radiological, and nuclear (CBRN) materials. The design concept of the CBRN transport capsule aimed to make the patient feel more secure, to promote trust, protection, and a blue color, which represents a feeling of calm (İşbilir et al., 2018). As there is a potential for clusters of COVID-19 patients, the design of containment capsules for COVID-19 patients must involve foldable, easy to construct structures that are easily transportable. The materials of the capsule must be light, have anti-corrosion properties, and be rigid during use. The use of aluminum tubes as the main structural component is an alternative way to create the capsule (Iván Cifuentes et al., 2020). Due to cost issues and availability mass production of the transporting capsule using aluminum as the predominant material may not appropriate for some developing countries, especially in Thailand.

In this pandemic, we also have found no complete protective-assisted ventilating device that can be used during the pre-intubation period, while waiting for invasive mechanical ventilation, and following the intubation period. Medical personnel, including the nurse operating the AMBU bag, the physician who inserts the endotracheal tube, and anyone who stays within that unit, must necessarily potentially be exposed to secretions from the patient. Even though some institutes have used an intubation acrylic box covered the patient’s head during intubation, following the procedure exhaled and therefore infected air can spread. During the intubation period, airborne particle sizes of 0.3–2.5 μm can be decreased when using the sealed intubation box with suction, on the contrary, the erosol box solely showed an increase in 1.0–5.0 μm when compared with no device used (Simpson et al., 2020). Therefore, the objective of this article was to discuss the design and fabrication of an AMBU ventilator with a negative pressure headbox, incorporated with a negative pressure transporting capsule. This dual machine, which could provide a low-cost construction, ease of use, and easy to fabricate without a high level of technology, could then be used to protect medical personnel during periods of intubation as well as transport between units.

## Materials and Methods

This article was an experimental and pilot study. The tests of this dual machine also included continuous working, functions, battery endurance, and negative air pressure test. To prevent the spread of COVID-19, these anti-airborne system consists of three parts, including, a mechanical ventilator for the pre-intubation and intubation periods before using a standard mechanical ventilator, a negative pressure headbox for the post-intubation period, and finally a transporting capsule, which will be used during a patient’s transfer. All materials within the ventilating system were certified as medical-grade products to make sure that patients will not suffer from any allergic conditions. The details of mechanical systems are shown below;

### Automated AMBU bag Ventilator

**FIGURE 1:**
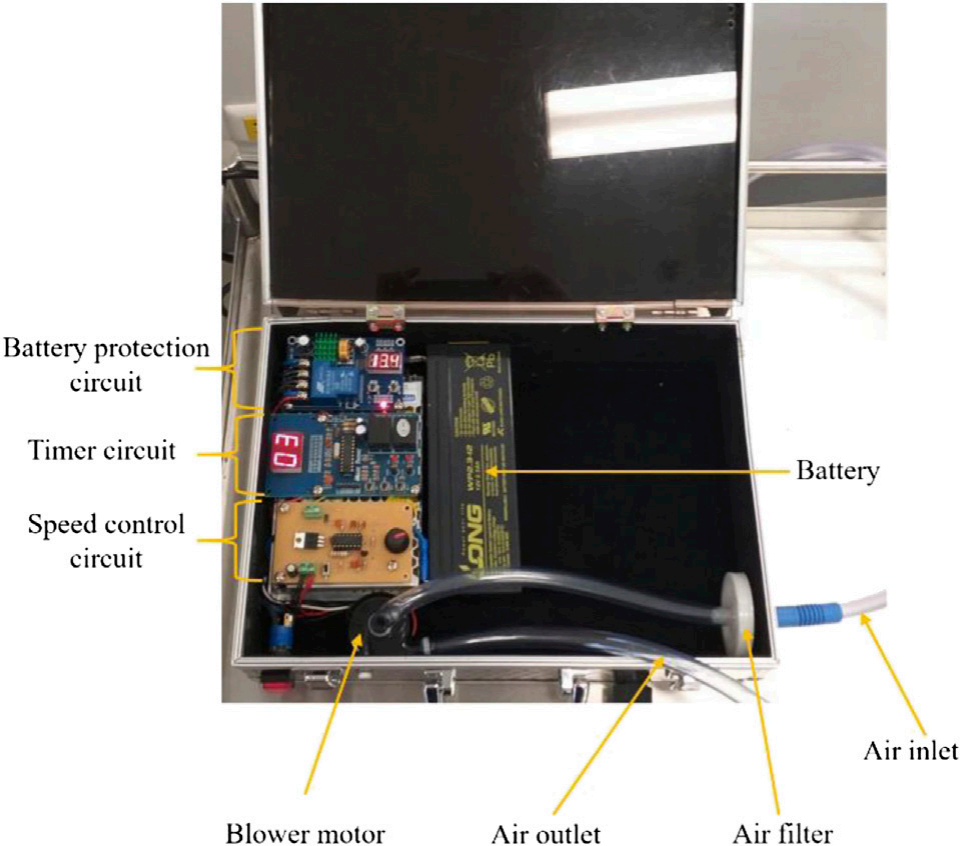
The prototype of the mechanical ventilator.

Oxygen within a pipeline or a tank initially passes through an air filter and then into the ventilating system. A 12 V DC brushless motor for medical equipment, max power consumption 18 W, was used as a blower motor. In general, according to standard respiratory physiology, the tidal volume, which means the volume of each respiration, is usually 500 ml with a rate of 12 breaths min^−1^ (Carroll, 2007). Therefore, mechanical ventilation should be able to be adjusted to both higher and lower levels than the standard volume and rate. To control flow rate, rhythm, and volume of oxygen, three electrical circuits, including, a speed control circuit, a timer circuit, and a battery protection circuit, were incorporated with the blower. A pressure release valve, set to operate if the air pressure exceeds 40 cm H_2_O, was fitted to the AMBU bag. A 12 V 2.3 Ah rechargeable sealed lead-acid battery was used as the main power supply. The machinery functions and working duration were tested. A mechanical ventilator prototype is shown in Figure 1.

### Negative Pressure Headbox

**FIGURE 2:**
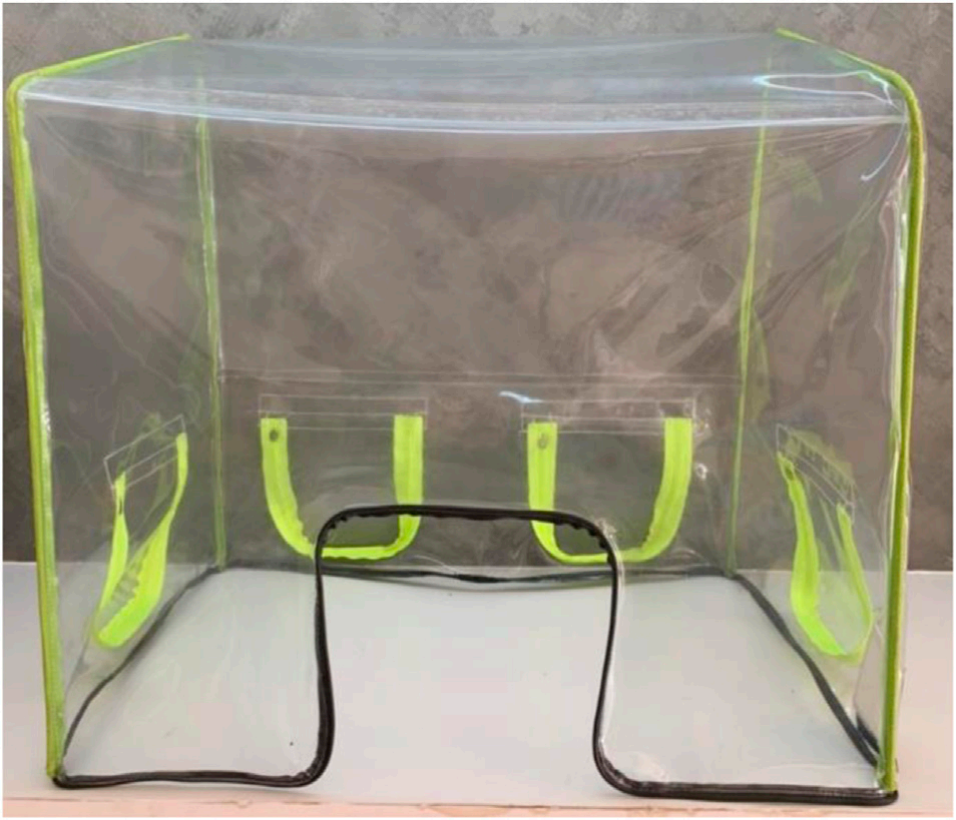
The prototype of the negative pressure headbox.

**FIGURE 3:**
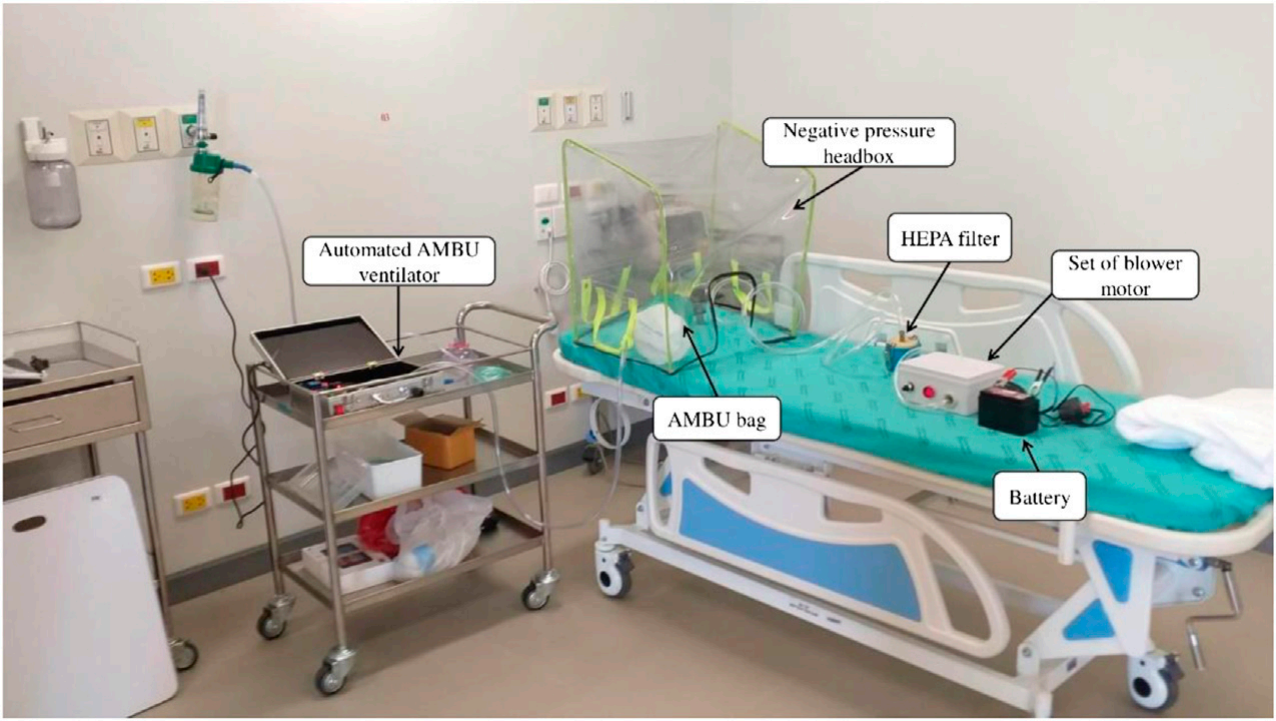
The prototype of the automated AMBU ventilator with the negative pressure headbox.

**FIGURE 4:**
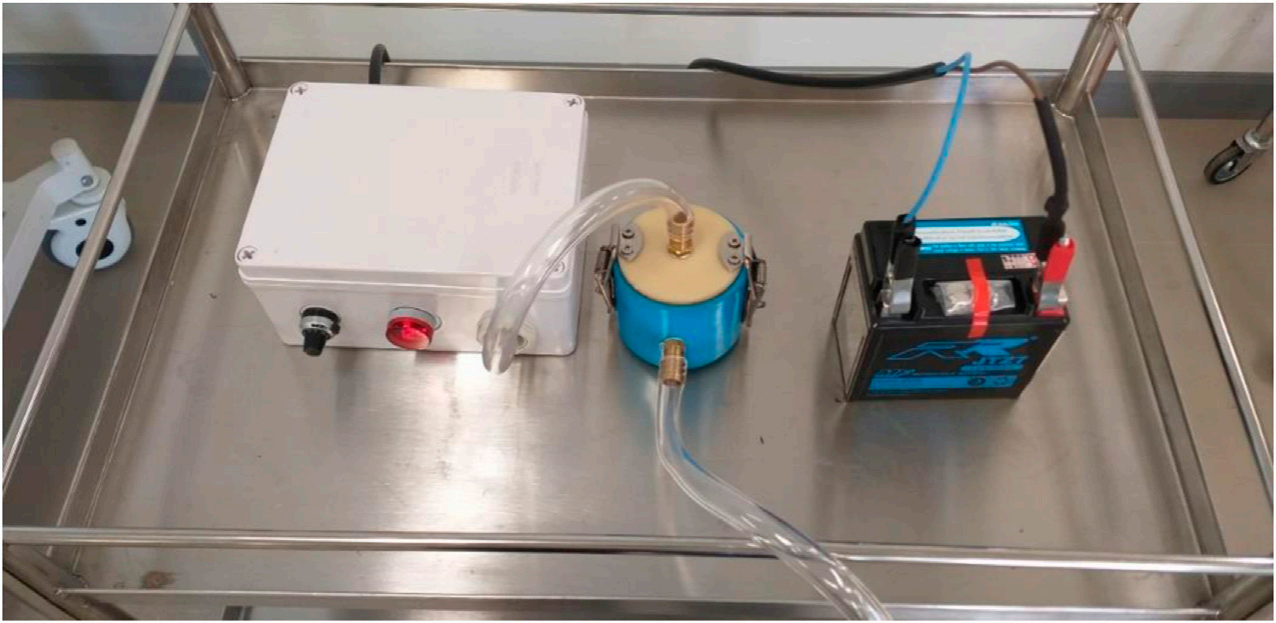
The set of blower motor within the box (left), HEPA filter (middle), and battery (right).

The headbox, which was mainly made of a PVC transparent sheet 0.5 cm thickness, was used as air-borne protective equipment. Four waterproof zippers were amalgamated with the headbox for hand and/or tube insertion. After the intubation procedure, hazardous air within the box was treated by HEPA filter and a blower motor that had the same specification as the mechanical ventilator. A 12 V 7 Ah lithium battery powered this machine. For ease of use all components of the headbox, except the HEPA filter, can be cleaned by any disinfectants. The blower and battery workings were tested. The negative pressure headbox model is shown in Figure 2 while the combination of an automated AMBU ventilator with a negative pressure headbox is shown in Figure 3. The set of HEPA filter, blower motor, and battery are shown in Figure 4.

### Negative Pressure Transporting Capsule

**FIGURE 5:**
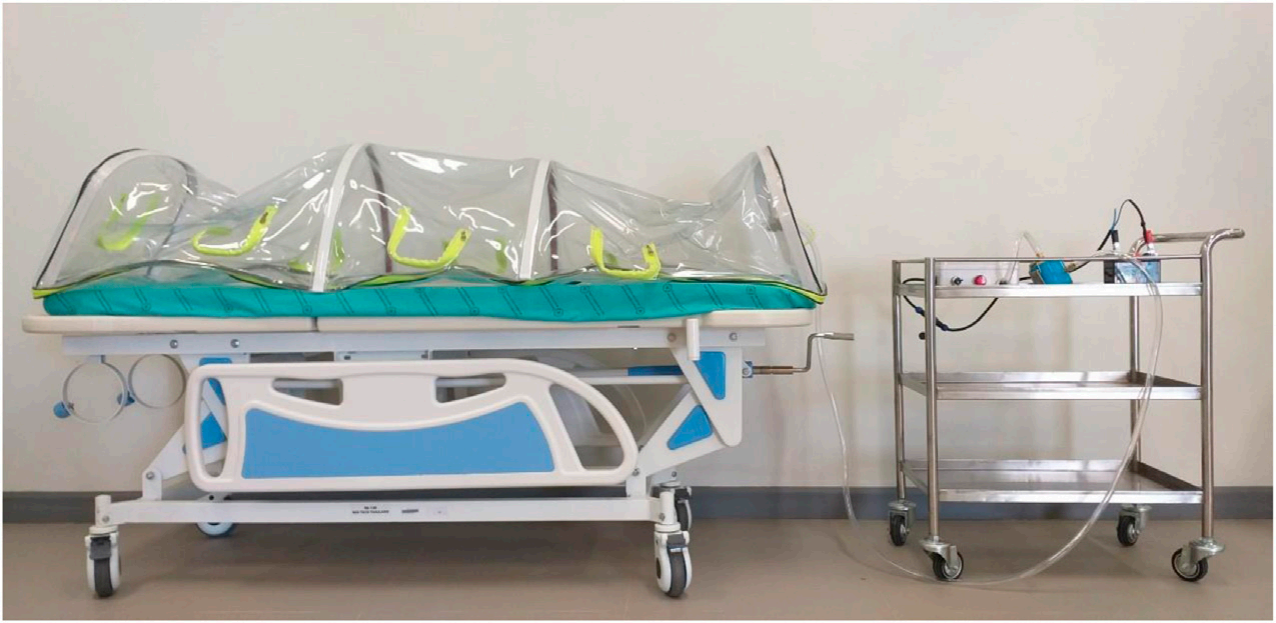
Model of the negative pressure transporting capsule with the set of the blower motor.

Similar with the negative pressure headbox, a semi-circular cylindrical transporting capsule, measuring 0.28 m^3^ in volume (where: r = 0.3 m and L = 2.0 m), which is shown in Figure 5, was made of PVC materials and waterproof zippers. The design of the capsule was of a compact size, which could be loaded into either an ambulance or CT scan. The set-up of the HEPA filter, blower motor, and the battery was the same as for the negative pressure headbox. A digital airflow anemometer Model GM8901, measurement range 0–45 m s^−1^ (accuracy ±3%), was used for air velocity measurement within the closed system. The calculation of air changes per hour used the following formula:

$$\mathrm{ACH}=\frac{3600\;\cdot Q}{\mathrm{Vol}}$$

Where:

• ACH = number of air changes per hour
• Q = volumetric flow rate of air in cubic meter per second
• Vol = space volume in a cubic meter

The pressure of the inside as well as outside capsule was measured using a portable multifunction digital LCD barometer that has a measurement range of 300 to 1,000 hPa.

According to the situation being, in Chiang Rai Province, Thailand, all COVID-19 patients have been sent to the Chiangrai Prachanukroh Hospital, which is the biggest medical center in Chiang Rai Province. By the way, no COVID-19 case has been detected at the Mae Fah Luang University Medical Center Hospital. We are therefore unable to test these instruments with COVID-19 patients at this moment. We will test the ventilator and capsule function absolutely in a real case as soon as possible. This step also only described how to fabricate and test in a preliminary laboratory scale. This experiment is only a design, fabrication, and test of machines without human participation. At this moment, the ethical committee review, therefore, was unnecessary.

## Results

**FIGURE 6:**
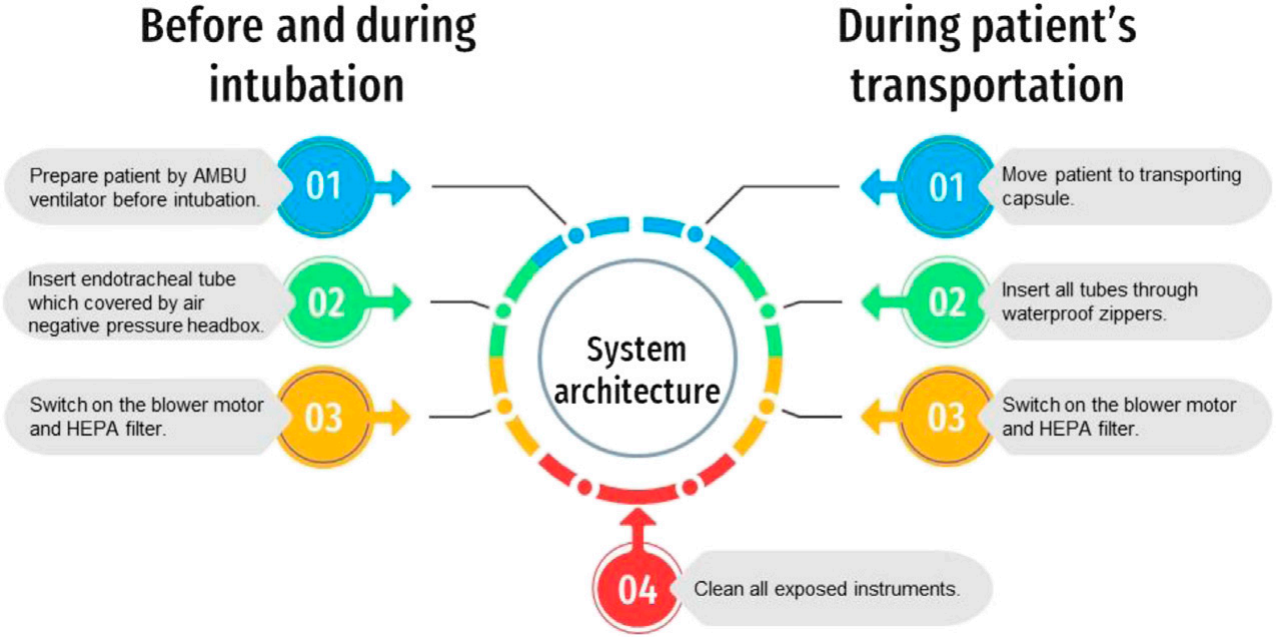
The system architecture diagram of the mechanical ventilator and the transporting capsule.

The dual prototype of an automated AMBU ventilator and transporting capsule was fabricated and tested. The preliminary outcomes showed that each mechanical system could work for more than 24 h continuously. The summary of the system architecture diagram of both machines is shown in Figure 6. The results are shown below.

### Functional and Endurance Test

The mechanical ventilator speed control circuit can be adjusted from 0 to 100%. The two main functions of the ventilator include, firstly, an active mode, which can be adjusted at the resolution of 1 s and can be set to cover a range of 0 s–9 h 59 min 59 s. The delay time of the blower motor is approximately 1 s. Secondly, a rest mode can be controlled as an active mode without delay time. At maximum power the blower filled a 2,000 ml reservoir bag within 1.5 s. It was confirmed that the battery system could operate for up to 2 h without recharging.

The blower motor and battery system, which were used to create negative air pressure within a headbox as well as the transporting capsule, were also able to run for at least 2 h without recharging, which is a sufficient time for transferring patient between units.

### Negative air Pressure Test

The transporting capsule was used for the negative air pressure experiment. The maximum air velocity at the end of the blower tube, measuring 1.905 cm in diameter, was 6 m s^−1^. Therefore, this capsule also showed 21.76 ACH, which was more than 2–3 times greater than that of the World Health Organization’s recommendations for such a system.

Air pressure within the capsule was approximately 10 Pa lower than the outside environment, when measured at 27.8°C, confirming that this method can create the required negative pressure for safe usage.

In case of any unforeseen issues, for example, if the mechanical ventilator cannot run, or maybe some accidents occur within the ventilation system, the user can pull the oxygen supply extension tubing off, then, connect it to the AMBU bag within the negative pressure headbox directly. The blower motor and the HEPA filter system of both the negative pressure headbox and the transporting capsule can be switched to another machine together. Therefore, medical personnel can use these air purifier systems simultaneously or individually.

## Discussion

All inventions that were fabricated in this study, were created within a time limit according to the COVID-19 situation. All development has been carried out at the Mae Fah Luang University Medical Center Hospital, Chiang Rai Province, Thailand, which is far away from the capital city. We have suffered with a lack of raw materials as well as monetary resources. These machines can run at least 24 h continuously. The mechanical ventilator can be used during pre-intubation and intubation period to control air flow rate, rhythm, and volume of oxygen in primary. The transporting capsule is of a compact size and incorporates the air treatment systems. Air pressure within the capsule was lower than the required negative pressure for safe usage. The hazardous expired air from the PVC headbox and also the transporting capsule was treated by a HEPA filter, which was able to run for at least 2 h without recharging. The author received a budget, to include the costs for fabrication and transportation, of 36,000 Thai Baht (THB) for the transporting capsule as well as 60,000 THB for the automated ventilator. As part of the ongoing development of the AMBU ventilator, a microcontroller should be incorporated to feedback any signals from the patient. For example, respiratory rate, positive end-expiratory pressure (PEEP), oxygen fraction, inspired tidal volume, expired tidal volume, peak pressure, etc. Then all parameters should be expressed on an LCD monitor and evaluated to provide optimum ventilation for each patient (Govoni et al., 2012).

A preliminary design of the transporting capsule focused on the overall utility of the machine, which should be able to be used within an ambulance or CT scanner. However, as a result of the COVID-19 travel restriction and state quarantine policies of Thailand, the inventor could not source suitable plastic materials to create the frame of this capsule. As a result thin wire had to be substituted to preserve the shape of the capsule. Other parts of the capsule that also had to be made from metal, were the waterproof zippers. In a second prototype it should be made of 100% plastic-based materials, without metal components, to allow compatibility of use with any diagnostic imaging devices. All devices should be able to be stored within a compact unit, so be portable and foldable.

Finally, these machines were fabricated and tested during the rush hour of the COVID-19 pandemic, they were also declared as the prototype scale. When the prototype concepts are finalized, they will be certified by national standard organizations (e.g., Thai Food and Drug Administration, ISO 13485, ISO 15189) and then testing of the machines with human subjects will commence.

## Conclusion

In summary, the automated AMBU ventilator allows the initial setting of preliminary flow rate, rhythm, and volume of oxygen during the intubation period, so protecting medical colleagues from potential erosol infection, but this first version has no ability to respond to respiratory feedback from the patients. The hazardous expired air is collected within the transparent PVC headbox and filtered through a HEPA filter, amalgamated with a blower motor, so returning fresh air to the environment. After intubation, patients can be transferred within the transport capsule, which has a compact size and contains the air treatment systems, including the negative pressure headbox, all constructed of PVC based materials. These machines, except some electronic parts that are separated from the contaminated airway systems and also have never exposed to viruses, can be cleaned by the same disinfectants as previous devices, for example, ethyl alcohol, sodium hypochlorite solution, calcium hypochlorite solution, hydrogen peroxide, etc. However, heat sterilization should be avoid because almost all headbox and also capsule materials were made of PVC sheet. Only the set box for housing HEPA filter can be treated using hot steam. This dual machine is safe, and can be fabricated easily in low and middle-income countries while the traditional ventilator systems also need some advanced technologies, followed by high-priced machines as well as the huge customary transport capsules that have been usually designed for transferring the patients between units, but also they cannot be entered into either an ambulance or CT scan. In the future all metal parts need to be replaced with plastic-based materials, operating standards need to be verified, testing on human subjects must be completed, and then the system needs to be properly marketed.

## Data Availability Statement

The original contributions presented in the study are included in the article/Supplementary Material, further inquiries can be directed to the corresponding author.

## Author Contributions

The author confirms being the sole contributor of this work and has approved it for publication.

## Funding

These inventions are supported by the Princess Srinagarindra’s Centenary Celebrations Foundation, Memorandum No. 28/2563 as well as the Mae Fah Luang Intellectual Property Management and Innovation Division, fiscal year 2020.

## Conflict of Interest

The author declares that the research was conducted in the absence of any commercial or financial relationships that could be construed as a potential conflict of interest.
